# Pancreatic adenocarcinoma in type 2 progressive familial intrahepatic cholestasis

**DOI:** 10.1186/1471-230X-10-30

**Published:** 2010-03-13

**Authors:** Lee M Bass, Deepa Patil, M Sambasiva Rao, Richard M Green, Peter F Whitington

**Affiliations:** 1 Northwestern University Feinberg School of Medicine, Department of Pediatrics, Chicago, Illinois, USA; 2 Northwestern University Feinberg School of Medicine, Department of Pathology, Chicago, Illinois, USA; 3 Northwestern University Feinberg School of Medicine, Department of Medicine, Chicago, Illinois, USA

## Abstract

**Background:**

BSEP disease results from mutations in ABCB11, which encodes the bile salt export pump (BSEP). BSEP disease is associated with an increased risk of hepatobiliary cancer.

**Case Presentation:**

A 36 year old woman with BSEP disease developed pancreatic adenocarcinoma at age 36. She had been treated with a biliary diversion at age 18. A 1.7 × 1.3 cm mass was detected in the pancreas on abdominal CT scan. A 2 cm mass lesion was found at the neck and proximal body of the pancreas. Pathology demonstrated a grade 2-3 adenocarcinoma with invasion into the peripancreatic fat.

**Conclusions:**

Clinicians should be aware of the possibility of pancreatic adenocarcinoma in patients with BSEP disease.

## Background

Type 2 Progressive Familial Intrahepatic Cholestasis (PFIC-2), also known as BSEP disease, is one of the genetically determined cholestatic diseases and results from mutations in ABCB11, which encodes the bile salt export pump (BSEP) protein responsible for the bulk of conjugated bile salt transport from hepatocytes into biliary canaliculi. BSEP disease has an estimated incidence in Western Europe of approximately 1 in 50,000-70,000 births [1]. While in most ways the clinical features of PFIC-2 are similar to PFIC-1 (FIC1 disease due to mutations in ATP8B1), it is uniquely associated with substantially increased risk of hepatobiliary cancer. Early presentation of hepatocellular carcinoma (HCC) was reported in 10 children with BSEP disease [2], and cholangiocarcinoma, a very rare cancer in children, was reported in 2 cases [3]. In a large survey of families with severe BSEP disease, 15% of patients developed HCC or cholangiocarcinoma [1]. This suggests that BSEP disease carries a HCC risk somewhat equivalent to that of tyrosinemia type 1, among the highest for any genetic disease [4], and also a risk for cancer involving other hepatic cell types, not seen in tyrosinemia. We describe a woman with BSEP disease who developed Pancreatic Adenocarcinoma and propose that this should be added to the cancer risk imposed by BSEP disease. This is the first reported case of pancreatic adenocarcinoma in a person with BSEP disease.

## Case Presentation

This female patient with BSEP disease presented at age 36 years for evaluation of a pancreatic mass. The mutation in her ABCB11 gene involves heterozygote missense mutations, one allelic mutation being 1238T>G (Leu413Trp) and the other 3724C>A (Gly1242Ile), as reported elsewhere [1]. She was diagnosed at age 18 with low-GGT PFIC when she presented with unremitting cholestasis with severe pruritus. Her older male sibling died prior to her birth at age 4 from cholestatic liver disease and HCC. Her family history also included pancreatic cancer in an uncle, colon cancer in two uncles, and liver cancer in the maternal grandfather. She underwent partial external biliary diversion for treatment of cholestasis, as reported elsewhere [5]. Her serum bile salts at diagnosis were >300 μmol/ml (<10 μmol/ml) and normalized following biliary diversion. She required treatment with ursodeoxycholic acid (10 mg/kg/d) to maintain full relief from pruritus. With this treatment, she was able to complete college and to work as an accountant. She did not use tobacco. At ages 26 and 29, she had successful pregnancies resulting in healthy unaffected children. However, with each of these pregnancies she experienced prolonged, severe cholestasis with jaundice and itching. The second of the two episodes was so severe that it prompted an extensive evaluation for possible contributing factors. Endoscopic retrograde cholangiography demonstrated patency of the external biliary diversion, and liver biopsy demonstrated canalicular cholestasis, focal loss of bile ducts and focal bridging fibrosis.

**Figure 1:**
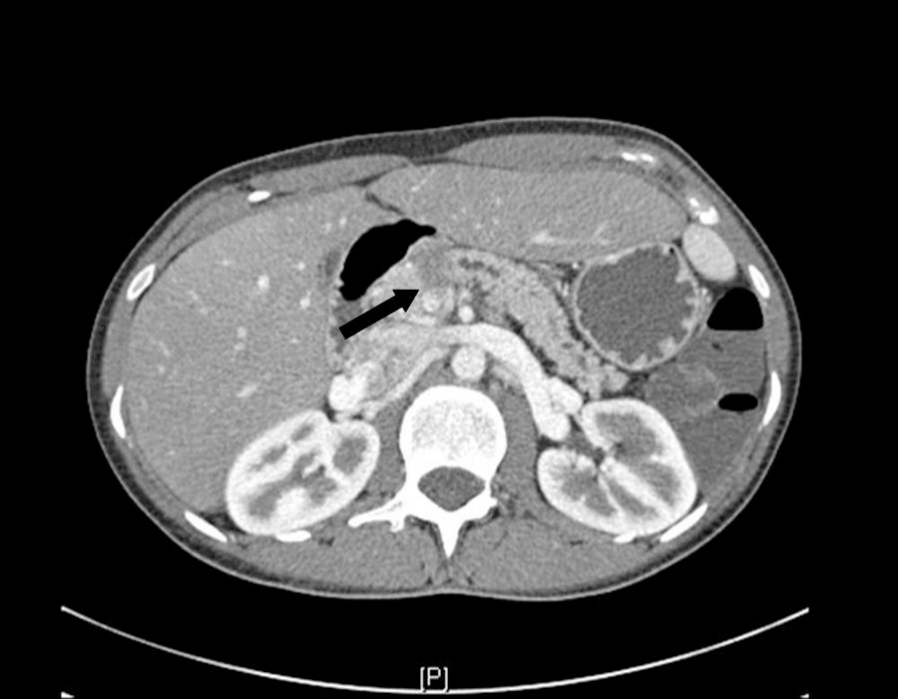
**CT scan of abdomen with pancreatic mass (arrow)**.

**Figure 2:**
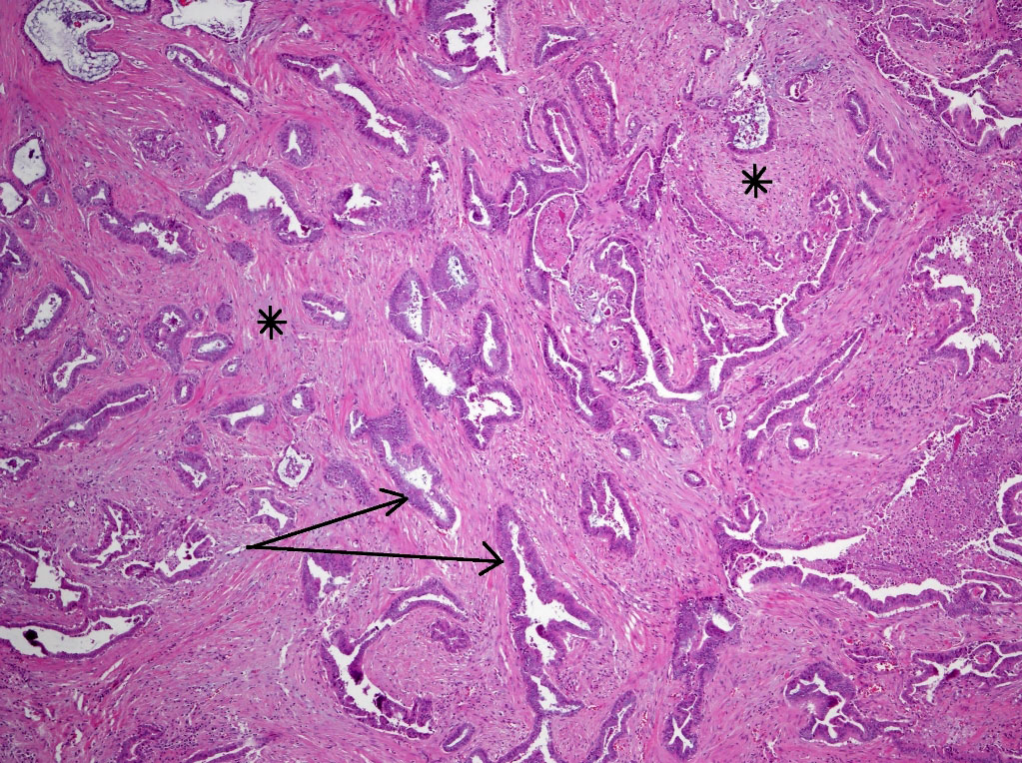
**Low magnification of pancreatic mass showing an infiltrative tumor composed of angulated glands (see arrow) distributed irregularly in a desmoplastic stroma (asterisk)**.

**Figure 3:**
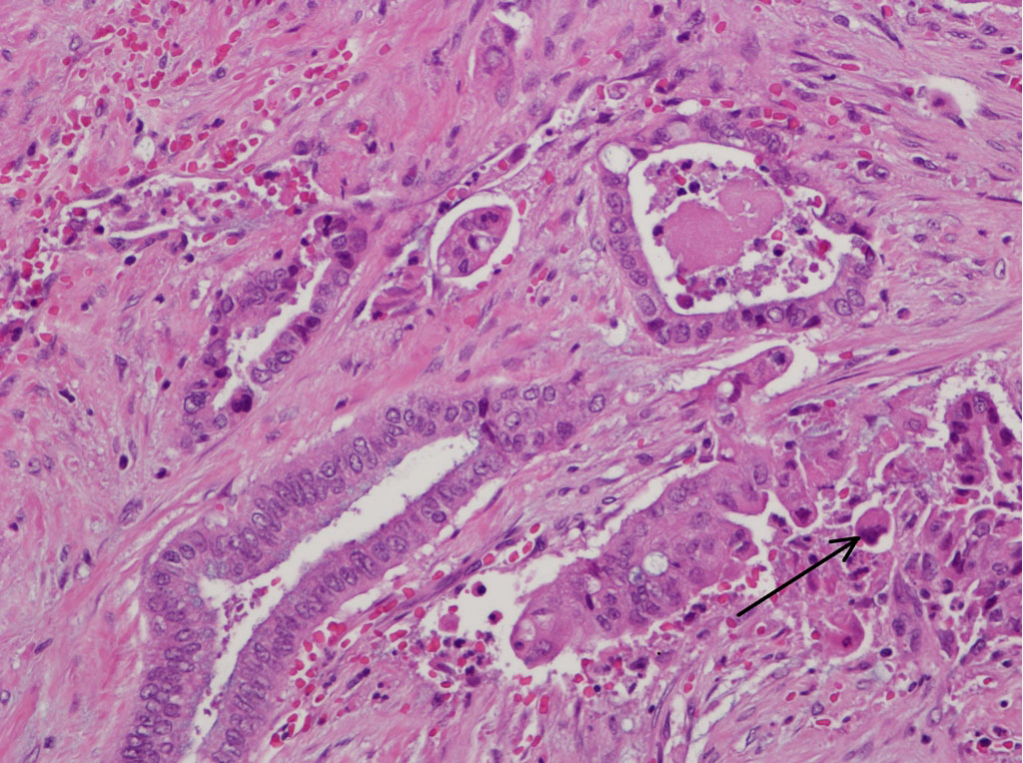
**Higher magnification of the tumor shows tumor glands lined by cells with abundant eosinophilic cytoplasm with enlarged, hyperchromatic nuclei**. While most of the tumor was a grade 2 adenocarcinoma, foci of single atypical cells (see arrow) infiltrating the pancreatic parenchyma, consistent with a poorly differentiated component, was also present.

While being screened twice yearly for hepatocellular carcinoma, a low-density enhancing mass lesion of the pancreas was identified on abdominal CT scan measuring 1.7 × 1.3 cm with dilatation of the pancreatic duct within the body and tail upstream from the lesion (Figure 1). No other focal mass lesions were identified in the head, body or tail of the pancreas. Endoscopic ultrasound-guided biopsy demonstrated clusters of epithelial cells with atypia and mucin production. The CEA level was 6.1 ng/ml (0-3 ng/ml) and CA 19-9 level was 14.9 U/ml (<33 U/ml). At surgery a 2 cm mass lesion was found at the neck and proximal body of the pancreas with no gross extension into the portal vein or hepatic artery. An extended distal pancreatectomy with resection of the neck and proximal body of the pancreas was performed. Sections from the pancreatic mass demonstrated an infiltrative tumor composed of angulated glands (See arrow, Figure 2) elicting a desmoplastic stromal response (Figure 2, asterisk). The glands were lined by cells with abundant eosinophilic cytoplasm with pleomorphic nuclei, diagnostic of pancreatic adenocarcinoma. While the predominant part of the tumor was moderately differentiated, foci of single atypical cells infiltrating the pancreatic parenchyma (Figure 2), consistent with a poorly differentiated component were also noted. (Figure 3, arrow). The tumor exhibited invasion into peripancreatic fat and metastasis was indentified in 2 out of 25 resected lymph nodes. Pancreatic Intraepithelial Neoplasia 1A and 1B were also present. Following recovery from surgery, she underwent radiation therapy and chemotherapy with Gemcitabine and 5-FU. She remained clinically well until age 38 when she developed rapid progression of liver disease with ascites, spider telangiectasias, worsening jaundice and evidence of portal hypertension. Further evaluation demonstrated an intrahepatic mass thought to be a HCC and multiple pulmonary nodules that were positive for adenocarcinoma. A TIPS procedure was performed for control of portal hypertension, but she developed sepsis and died soon thereafter.

## Conclusions

Chronic cholestasis in children is often the result of genetic disease. Mutations in ATP8B1 (encoding for FIC1) and ABCB11 (encoding for BSEP) are the common causes of PFIC that are associated with relatively low serum gamma-glutamyl transferase (GGT) activity, so called low-GGT PFIC. They are very similar in presentation and both may respond to bile diversion therapy. However, patients with BSEP disease appear to have much greater risk for hepatobiliary cancer than do those with FIC1 disease. This case suggests that pancreatic cancer should be added to the list of cancers these patients are at potential risk for developing. We can only speculate on why this BSEP disease patient developed early onset pancreatic adenocarcinoma.

Pancreatic cancer is the third most common gastrointestinal malignancy in the United States. In 2007, more than 37,000 new cases were diagnosed in the US. The prognosis is poor: the 1 year survival is 26% and the 5 year survival rate 5%. Pancreatic cancer is the fourth leading cause of cancer related death in the US. Even in patients diagnosed with local disease, the 5 year survival rate is 20% [6]. The woman reported herein is unusual among pancreatic cancer cases for several reasons. First, this condition is relatively rare in younger persons. Only about 10% of cases are diagnosed before age 50 [7]. Secondly, it is somewhat more prevalent in males. Despite the odds against it, this case may represent only coincidence of two very rare conditions in one unfortunate woman.

Familial risk factors unrelated to BSEP disease may have placed her at increased risk. The patient had a sibling with BSEP disease as well as several family members apparently without BSEP disease who developed cancer. This family history may imply a genetic risk factor for malignancy above and beyond BSEP disease although the family members may be heterozygotes for the BSEP mutation. Risk factors in early onset pancreatic cancer include family history and cigarette smoking [8], which this patient did not do. Genetic mutations in pancreatic cancer have included loss of function of tumor suppressor genes CDKN2A/p16, p53 and LKB1/STK11 [9,10]. Mutant KRAS2, aberrant DNA methylation, and inactivation of TP53 and SMAD4 have been implicated [11]. BRCA2 gene mutations are also common [12].

Exposure to bile acids may offer another explanation for development of pancreatic adenocarcinoma in this patient. Xiao and colleagues have found that epithelial cells in liver cirrhosis exhibit features of hepatic stem cells [13]. Damage induced by intrahepatic bile acids can induce disorderly proliferation of stem cell elements that can differentiate along biliary epithelial lines [14], perhaps predisposing to intrahepatic cholangiocarcinoma. Scheimann and colleagues report 2 cases of cholangiocarcinoma in the setting of BSEP deficiency, both in the setting of intrahepatic cholestasis, fibrosis and inflammation [3]. It is conceivable that the exposure of pancreas cells to bile acids in our patients might also predispose to damage leading to increased carcinogenesis. It is of interest that the development of pancreatic cancer followed a period of protracted cholestasis related to pregnancy whereas she had been free of cholestasis for over a decade before. In opposition to this theoretical mechanism, Lu and colleagues have demonstrated that the growth of some pancreatic cancer cell lines is inhibited by bile acids [15]. Furthermore, there is no significant difference in the degree of cholestasis (i.e. degree of serum bile salt elevation) in FIC1 disease and other childhood cholestatic diseases such as Alagille syndrome as compared to BSEP disease. Finally, HCC has developed in BSEP patients after complete resolution of cholestasis and normalization of serum bile salt levels with bile diversion therapy [2]. Thus, the toxicity of bile salts in tissues and organs remote to hepatocytes as a cause for increased cancer risk in BSEP disease seems unlikely.

The hypothesis most likely explaining the development of pancreatic adenocarcinoma in our patient involves BSEP itself. BSEP may act as a tumor suppressor gene, and its malfunction may predispose to oncogenesis. Knisely and colleagues were the first to describe the apparent high incidence of HCC in children with BSEP deficiency. Eight of the 10 children had elevated serum concentrations of AFP, and AFP was demonstrated in the cytoplasm in all tumors. Nuclei in 8 of the tumors stained for p53. β catenin accumulation was not detected in any of the livers [2]. Strautnieks and colleagues identified 82 different ABCB11 mutations in 109 families with severe BSEP deficiency. Of affected patients, 15% had HCC or cholangiocarcinoma. The relative risk for cancer of two particular protein truncating mutations was significant, but the risk for malignancy was still 10% in patients without protein truncating defects [1]. Thus, at least in regard to HCC and cholangiocarcinoma, the ABCB11 gene defect appears to be an independent risk factor.

FIC1 is expressed in many tissues, most intensely in bowel and liver, and FIC1 disease patients have disease outside the liver including diarrhea, loss of hearing, and pancreatic insufficiency [16]. In contrast BSEP is expressed primarily in the liver. Thus, BSEP disease has few if any primary extrahepatic manifestations, and exocrine pancreatic function is thought to be normal [17]. Despite the fact that hepatocytes are the only cells in the body that have BSEP expression [18], there is embryologic evidence for homology in pancreas and liver cells. The endoderm of the ventral lateral foregut is induced into forming pancreas and liver by interaction with the cardiogenic mesoderm and expression of the homeobox gene Hex. Mice deficient in Hex have failure of ventral pancreas expression [19]. The pancreas emerges independently from dorsal and ventral domains of embryonic gut endoderm. The liver and ventral pancreas are specified at the same time and in the same general domain of cells. In embryo tissue explantation experiments in mice using ventral foregut endoderm, it was found that the default fate of the ventral foregut endoderm is to activate the pancreatic gene program. However, if exposed to Fibroblast Growth Factor signaling from the cardiac mesoderm, the ventral foregut endoderm expresses genes for the liver [20]. Additionally, the gene Hes 1 is a potential controller for the differentiation of pancreas and biliary epithelium. Hes1 knock out mice have agenesis of the gallbladder, hypoplastic extrahepatic bile ducts and biliary epithelium that differentiates into endocrine and exocrine like pancreatic structures [21]. Thus, the cells of the ventral foregut endoderm are pluripotential cells for the development of both pancreas and liver. While it is unclear at which point in organ development BSEP expression starts, it can be inferred based on the origin of the cells that mutated BSEP may have an impact on the pancreas. Thus the expression pattern of BSEP is not supportive of this hypothesis, but it remains possible that mutated BSEP may predispose to the development of pancreatic adenocarcinoma.

In summary, this is the first case report describing pancreatic adenocarcinoma in a patient with BSEP deficiency. Recommendations already exist in this disorder for screening of patients for HCC [2]. It stands to reason that liver transplant would not replace defective BSEP in the other cells of the body, despite normal liver function. Thus, patients may remain at risk for this pancreatic cancer despite successful liver transplantation. As the known population with this disorder ages, clinicians should be aware of the possibility of pancreatic adenocarcinoma in patients with BSEP deficiency.

## Abbreviations

BSEP: Bile Salt Export Pump; HCC: Hepatocellular Carcinoma; PFIC: Progressive Familial Intrahepatic Cholestasis.

## Competing interests

All of the authors certify that they have no commercial associations or others that pose a conflict of interest in connection with the submitted article.

## Authors' contributions

LB drafted the manuscript of the paper. MR and DP interpreted pathology findings and provided microscopic images for the manuscript. RG and PW participated in the care of the patient and assisted in drafting the manuscript. All authors read and approved the final manuscript

## Pre-publication history

The pre-publication history for this paper can be accessed here:

http://www.biomedcentral.com/1471-230X/10/30/prepub

## References

[B1] Strautnieks SS Byrne JA Pawlikowska L Cebecauerova D Rayner A Dutton L Meier Y Antoniou A Stieger B Arnell H Severe bile salt export pump deficiency: 82 different ABCB11 mutations in 109 families Gastroenterology 2008 134 4 1203 1214 10.1053/j.gastro.2008.01.038 18395098

[B2] Knisely AS Strautnieks SS Meier Y Stieger B Byrne JA Portmann BC Bull LN Pawlikowska L Bilezikci B Ozcay F Hepatocellular carcinoma in ten children under five years of age with bile salt export pump deficiency Hepatology 2006 44 2 478 486 10.1002/hep.21287 16871584

[B3] Scheimann AO Strautnieks SS Knisely AS Byrne JA Thompson RJ Finegold MJ Mutations in bile salt export pump (ABCB11) in two children with progressive familial intrahepatic cholestasis and cholangiocarcinoma J Pediatr 2007 150 5 556 559 10.1016/j.jpeds.2007.02.030 17452236

[B4] Weinberg AG Mize CE Worthen HG The occurrence of hepatoma in the chronic form of hereditary tyrosinemia J Pediatr 1976 88 3 434 438 10.1016/S0022-3476(76)80259-4 173827

[B5] Emond JC Whitington PF Selective surgical management of progressive familial intrahepatic cholestasis (Byler's disease) J Pediatr Surg 1995 30 12 1635 1641 10.1016/0022-3468(95)90440-9 8749912

[B6] Jemal A Siegel R Ward E Murray T Xu J Thun MJ Cancer statistics, 2007 CA Cancer J Clin 2007 57 1 43 66 10.3322/canjclin.57.1.43 17237035

[B7] Lowenfels AB Maisonneuve P Epidemiology and risk factors for pancreatic cancer Best Pract Res Clin Gastroenterol 2006 20 2 197 209 10.1016/j.bpg.2005.10.001 16549324

[B8] Raimondi S Maisonneuve P Lohr JM Lowenfels AB Early onset pancreatic cancer: evidence of a major role for smoking and genetic factors Cancer Epidemiol Biomarkers Prev 2007 16 9 1894 1897 10.1158/1055-9965.EPI-07-0341 17855711

[B9] Ruggeri BA Huang L Berger D Chang H Klein-Szanto AJ Goodrow T Wood M Obara T Heath CW Lynch H Molecular pathology of primary and metastatic ductal pancreatic lesions: analyses of mutations and expression of the p53, mdm-2, and p21/WAF-1 genes in sporadic and familial lesions Cancer 1997 79 4 700 716 10.1002/(SICI)1097-0142(19970215)79:4<700::AID-CNCR7>3.0.CO;2-H 9024708

[B10] Frank SA Age-specific incidence of inherited versus sporadic cancers: a test of the multistage theory of carcinogenesis Proc Natl Acad Sci USA 2005 102 4 1071 1075 10.1073/pnas.0407299102 15657129 PMC545832

[B11] Brune K Hong SM Li A Yachida S Abe T Griffith M Yang D Omura N Eshleman J Canto M Genetic and epigenetic alterations of familial pancreatic cancers Cancer Epidemiol Biomarkers Prev 2008 17 12 3536 3542 10.1158/1055-9965.EPI-08-0630 19064568 PMC2664523

[B12] Couch FJ Johnson MR Rabe KG Brune K de Andrade M Goggins M Rothenmund H Gallinger S Klein A Petersen GM The prevalence of BRCA2 mutations in familial pancreatic cancer Cancer Epidemiol Biomarkers Prev 2007 16 2 342 346 10.1158/1055-9965.EPI-06-0783 17301269

[B13] Xiao JC Ruck P Adam A Wang TX Kaiserling E Small epithelial cells in human liver cirrhosis exhibit features of hepatic stem-like cells: immunohistochemical, electron microscopic and immunoelectron microscopic findings Histopathology 2003 42 2 141 149 10.1046/j.1365-2559.2003.01544.x 12558746

[B14] Nomoto K Tsuneyama K Cheng C Takahashi H Hori R Murai Y Takano Y Intrahepatic cholangiocarcinoma arising in cirrhotic liver frequently expressed p63-positive basal/stem-cell phenotype Pathol Res Pract 2006 202 2 71 76 10.1016/j.prp.2005.10.011 16377099

[B15] Lu yO M Uchida E Yamamura S Yanagi K Matsushita A Kobayashi T Fukuhara M Aida K Tajiri T The Cytotoxic Effects of Bile Acids in Crude Bile on Human Pancreatic Cancer Cell Lines Surg Today 2000 30 903 909 10.1007/s005950070042 11059730

[B16] Lykavieris P van Mil S Cresteil D Fabre M Hadchouel M Klomp L Bernard O Jacquemin E Progressive familial intrahepatic cholestasis type 1 and extrahepatic features: no catch-up of stature growth, exacerbation of diarrhea, and appearance of liver steatosis after liver transplantation J Hepatol 2003 39 3 447 452 10.1016/S0168-8278(03)00286-1 12927934

[B17] Walkowiak J Jankowska I Pawlowska J Strautnieks S Bull L Thompson R Herzig KH Socha J Exocrine pancreatic function in children with progressive familial intrahepatic cholestasis type 2 J Pediatr Gastroenterol Nutr 2006 42 4 416 418 10.1097/01.mpg.0000218154.26792.6a 16641580

[B18] Knisely AS Progressive familial intrahepatic cholestasis: a personal perspective Pediatr Dev Pathol 2000 3 2 113 125 10.1007/s100240050016 10679031

[B19] Bort R Martinez-Barbera JP Beddington RS Zaret KS Hex homeobox gene-dependent tissue positioning is required for organogenesis of the ventral pancreas Development 2004 131 4 797 806 10.1242/dev.00965 14736744

[B20] Deutsch G Jung J Zheng M Lora J Zaret KS A bipotential precursor population for pancreas and liver within the embryonic endoderm Development 2001 128 6 871 881 11222142 10.1242/dev.128.6.871

[B21] Sumazaki R Shiojiri N Isoyama S Masu M Keino-Masu K Osawa M Nakauchi H Kageyama R Matsui A Conversion of biliary system to pancreatic tissue in Hes1-deficient mice Nat Genet 2004 36 1 83 87 10.1038/ng1273 14702043

